# Serotype and genotype analysis of dengue virus by sequencing followed by phylogenetic analysis using samples from three mini outbreaks-2007-2009 in Pakistan

**DOI:** 10.1186/1471-2180-11-200

**Published:** 2011-09-10

**Authors:** Zareen Fatima, Muhammad Idrees, Mohammad A Bajwa, Zarfishan Tahir, Obaid Ullah, Muhammad Q Zia, Abrar Hussain, Madiha Akram, Bushra Khubaib, Samia Afzal, Saira Munir, Sana Saleem, Bisma Rauff, Sadaf Badar, Mahrukh Naudhani, Sadia Butt, Mahwish Aftab, Liaqat Ali, Muhammad Ali

**Affiliations:** 1 Division of Molecular Virology, CEMB University of the Punjab, 87-West Canal Bank Road, Thokar Niaz Baig, Lahore-53700, Pakistan; 2 Department of Gastroenterology, Sheikh Zayed Medical Complex, Lahore, Pakistan; 3 Bacteriologist Laboratory, Institute of Public Health, 6-Birdwood Road, Lahore, Pakistan

## Abstract

**Background:**

Since the first reported outbreak of dengue hemorrhagic fever in Pakistan, several mini outbreaks have erupted in the region. Dengue virus serotype 3 (DEN-3) was first documented in 2005 outbreak in Karachi. Reports show that serotype 3 is prevalent in Lahore since 2008. Serotype 2 (DEN-2) is the major circulating serotype in Pakistan as it is documented since 1994. We have conducted a detailed study of three outbreaks of dengue virus infection that occurred in years 2007, 2008 and 2009 in Lahore by using molecular techniques such as PCR and nucleotide sequencing of the *C-prM *gene junction of Dengue virus.

**Results:**

Through the analysis of 114 serum samples collected over the period of three years (2007-2009), total 20 patients were found to be infected with dengue virus. In year 2007, four were positive for serotype 2 and one sample was positive for serotype DEN-3. In 2008, five samples had concurrent infection with serotypes DEN-2 and DEN-3 while three samples were infected only with serotype DEN-2. In year 2009, one sample had concurrent infection with serotypes DEN-2 and DEN-3 while six were positive for serotype DEN-2 only.

**Conclusions:**

Our study showed that serotype DEN-2 was dominant in positive samples of dengue virus infection collected during the period of three years (2007-2009). The other serotype present was serotype DEN-3. Genotypes of serotype DEN-2 and serotype DEN-3 were subtype IV and subtype III, respectively.

## Background

Dengue infection is an important mosquito-borne viral infection in areas where mosquitoes breed under optimal conditions. As a member of the family Falviviridae, the dengue virus is transmitted to human via *Aedes *genus, especially *Aedes agypti*. This family also includes Hepatitis C Virus, West Nile Virus and Yellow Fever Virus. Dengue virus has four serotypes DEN 1-4. Sequencing of dengue viral RNA has further verified strain variation within a serotype allowing viruses to be classified into genetically distinct groups within serotypes called genotypes. This virus is prevalent in areas of Asia, Africa, Central and South America [1,2] . Dengue viral infection can either cause dengue fever (DF), dengue hemorrhagic fever (DHF) or dengue shock syndrome (DSS). The classical dengue fever is mild, febrile illness which usually results after primary infection with dengue virus. In other cases DF can lead to DHF or DSS which can be life threatening [3,4]. Infection with a different serotype can show severe outcome due to antibody dependent enhancement [2,5] and can be a risk factor for DHF and DSS [2,6-8]. Though dual infection with dengue virus is attributed to cause onset of severe disease [9-11] but a case of mild disease due to dual infection was documented in Brazil in 2003 [9]. Outcome of disease may also depend upon the genotype involved. Some genotypes induce greater viremia and are transmitted more readily, thereby having a higher potential to cause large epidemic [12,13].

Timely and correct diagnosis is very critical for patient management as no definitive vaccine has been developed against all dengue virus serotypes. Methods are being employed for diagnosing the dengue virus infection like viral isolation techniques, serological methods and molecular methods. Viral isolation methods are time consuming and usually take a week [2,14]. Use of serological methods by detecting viral anti-IgM anti-IgG can give false positive results due to extensive antigenic cross-reactivity among flavivirus as well as between different dengue virus serotypes [2,15-17]. Different types of polymerase chain reactions (PCR) like reverse -transcription PCR (RT-PCR), real-time PCR and nested or hemi-nested PCR are used for detecting genomic sequence for serotyping. Use of PCR techniques is a quick and sensitive method for detecting dengue virus and has replaced viral isolation techniques [2,18].

Several outbreaks due to the dengue virus infection have been reported from Pakistan [19-26]. Dengue infection was first documented in Pakistan in year 1982 from Punjab in which 12 patients out of total 174 were found positive for dengue virus; all these samples were collected in1968 and 1978 [19]. The first outbreak of DHF was documented in 1994 by Chan and colleagues [21] who observed DEN-1 and DEN-2 in three out of ten tested patients for dengue virus. In the following year, DEN-2 infection was reported from the province of Balochistan [22,23]. Through serological studies, dengue type 1 and type 2 were found in sera of children in Karachi [24,25]. Jamil and colleagues [20] had previously been reported DEN-3 infection in 2005 outbreak of DHF in Karachi. Kan and colleagues [26] reported co-circulation of dengue virus type 2 and type 3 in 2006 outbreak in Karachi. More recently, Hamayoun and colleagues [22] reported cases with dengue infection in the 2008 outbreak in Lahore. Out of 17 samples checked via real-time PCR, ten of their patients had DEN-4, five had DEN-2 and two had DEN-3 infection [22].

Pakistan has a history of outbreaks of dengue viral infection however, the responsible serotype/s is not well known. Therefore, the current study was initiated to determine the circulating serotype/s of dengue virus in Pakistan using molecular based techniques in patients' sera. Samples were selected from stored repository from three most recent outbreaks of dengue virus (2007-2009) and the obtained sequences were compared to other dengue virus sequences reported from other geographical regions of the world to deduce a phylogenetic relationship.

## Results

### Serotyping of analyzed sample

A total of 114 suspected dengue serum samples along with demographic data were kindly donated by Gurki Trust Hospital Lahore and Sheikh Zayed Medical Complex Lahore for the current study. These samples were collected during three different mini outbreaks of dengue virus infection in years 2007, 2008 and 2009 and were stored at -20°C. Nested PCR was utilized for this serotype analysis. Out of total 114 tested serum samples, 20 were found positive for dengue virus RNA with various serotypes. Table 1 shows the distribution of dengue virus serotypes in the study population. It is clear from the results of the current study that, of the 20 dengue virus positive samples, six had concurrent infection with two different dengue virus serotypes at a time generating data of 26 serotypes.

**Table 1:** Total positive samples and dengue virus isolates included in this study.

Year of isolation	Total collected samples	Positive samples	Isolated serotype*	
			
			Serotype 2	Serotype 3
2007	41	5	4	1
2008	66	8	8	5
2009	7	7	7	1

***Total***	**114**	**20**	**19**	**7**

*Out of 20 positive samples, 6 samples had concurrent infection with two dengue virus serotypes giving a total of 26 dengue virus isolates.

### Nucleotide sequences analysis

The amplified bands of each sample were gel eluted and were further used for sequence analysis. Junction of *C-prM *gene of dengue virus isolates was chosen for serotyping. Accession numbers of these 26 studied sequences are [GenBank: HQ385930-HQ385943 and HM626119-HM626130]. The length of amplified product was 403 base pairs (bp) for serotype 2 and 453 bp for serotype 3. The BLAST search was done and the sequences of serotype 2 were found close to a Sri Lankan strain [GenBank: GQ252676] with an average of 99% homology. The sequences of serotype 3 were close to a Chinese strain [GenBank: GU363549] with an average homology of 99%. These two strains were taken as prototypes for respective serotypes. The *C-prM *fragment of serotype 2 was found to be rich in AG composition with an average percentage of 32.7% and 25.4% respectively. The *C-prM *gene junction of serotype 3 was also found AG rich with an average percentage of 29.3% for A and 25.1% for G.

Further the obtained nucleotide sequences were translated using the BioEdit software. Translated results showed that amino acid tyrosine is not present in the polyprotein fragment of serotype 2. This region is rich in leucine with an average of 12.78% followed by arginine (10.64%). The polyprotein fragment of serotype 3 was found rich in leucine (12.58%) and lysine with an average of 10.67%.

### Multiple sequence alignment and phylogenetic analysis of the sequences

**Figure 1:**
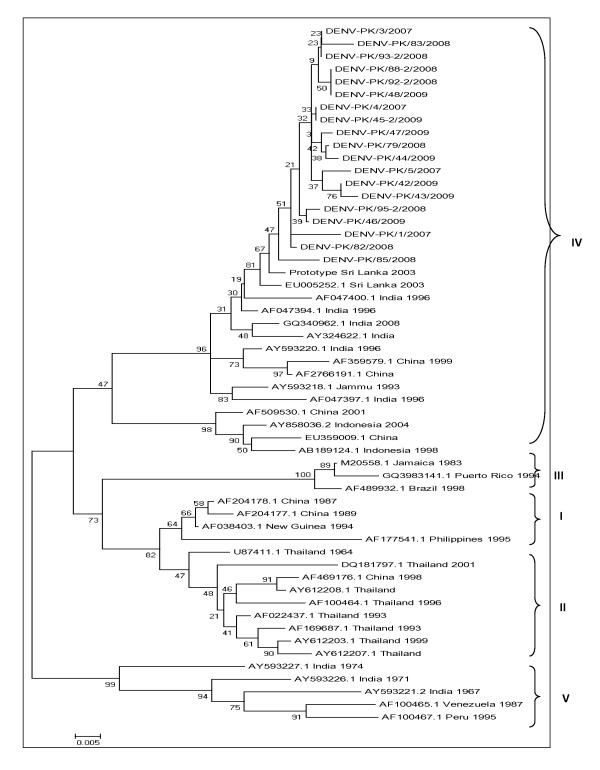
**Phylogenetic tree of studied sequences with geographical strains of serotype 2 generated by neighbor-joining method**. The tree is based on *C-prM *regions (nt194-522, 329 bp) of the selected strains. Each geographical strain is abbreviated by its accession number followed by country and year of isolation.

**Figure 2:**
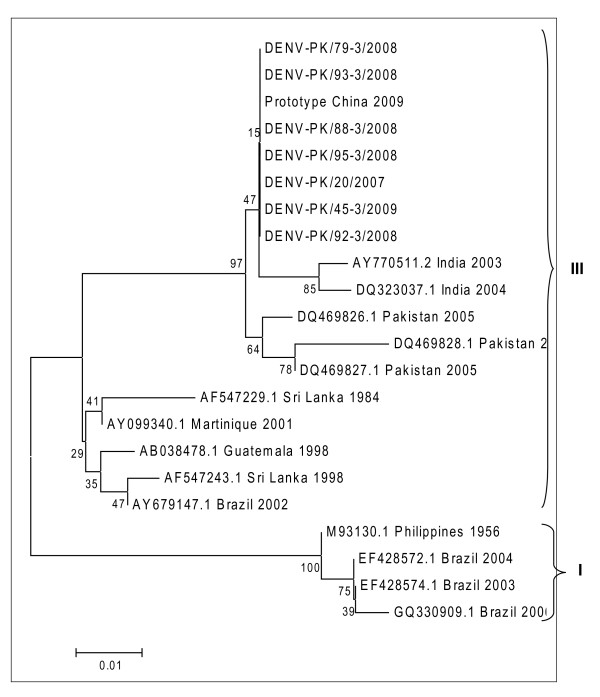
**Phylogenetic tree of studied sequences with geographical strains of serotype 3 by neighbor-joining method**. The tree is based on *C-prM *regions (nt200-418, 219 bp) of the selected strains. Each geographical strain is abbreviated by its accession number followed by country and year of isolation.

Phylogenetic tree was conducted using the MEGA 4 software and multiple sequence alignment was deduced by using BioEdit software. A region corresponding to nt122-523 (401-bp) of the prototype was aligned for sequences of serotype 2. Similarly region of nt158-609 (451-bp) was aligned for the sequences of serotype 3. Regions of both of the serotypes were not hyper variable. No insertions or deletions were seen in the regions of both serotypes. A slight variation in nucleotide sequences and translated polyprotein sequences was observed for sequences of serotype 2. The serotype 3 sequences were almost identical and same type of polyprotein was translated from the nucleotide sequences. Phylogenetic analysis was constructed among the sequenced isolates as well with different geographical isolates sequences. The sequences were retrieved from GenBank data base and 35 diverse sequences from different geographical regions were selected for serotype 2. For serotype 3, eleven sequences from different geographical regions of the world and 3 sequences from Pakistan were selected. A 329-bp region (nt194-522 of prototype 2) for serotype 2 and 219-bp region (nt200-418 of prototype-3) for serotype 3 was chosen. On constructing the tree, the sequenced serotype 2 lied in the category of genotype IV (Figure 1). The sequences fall in genotype IV with northern Indian strains. As there are no submitted sequences of genotype II and IV for capsid region of serotype 3, so the tree was constructed using sequences from genotype I and III. But the tree clearly showed that the studied sequences of serotype 3 had genotype III (Figure 2). They fall in the same genotype with Indian strains and other three Pakistani strains from Karachi.

## Discussion

Over the years dengue fever has become an important arboviral infection in different geographical regions of the world that supports the growth of mosquitoes. Its range exceeds over a hundred tropical and subtropical countries with more than 2.5 billion people at the risk of infection [12]. Pakistan has witnessed some severe outbreaks of dengue viral infection leading to significant morbidity and mortality since 1994 [20,21]. Since the publication of a study in 1982 documenting dengue infection from the years 1968 and 1978 [19], several mini outbreaks of dengue viral infection have been reported. No doubt all the four distinct serotypes, DEN-1, DEN-2, DEN-3, and DEN-4 of dengue virus have been reported as the cause of dengue infection; however, serotypes DEN-2 and DEN-3 remained the major cause of infection in humans world-wide. Like other parts of the world, in the current study we have observed that serotypes DEN-2 and DEN-3 are the predominant serotypes in dengue infection in outbreaks of 2007, 2008 and 2009 in Pakistan. In 2007, serotype DEN-2 prevailed with less occurrence of serotype DEN-3. In samples of 2008 and 2009, serotype DEN-3 has been isolated, though first incidence of serotype 3 infections was reported by Jamil and colleagues [20] in year 2005 outbreak in Karachi. This shows that serotype 3 is new comer to this region as was isolated for the first time in year 2005. All the previous outbreaks have been attributed to other serotypes. From Lahore, Hamayoun and colleagues [21] reported only serotype DEN-3 in 2008 outbreak and they were unable to isolate any other serotype. This finding of Hamayoun [21] confirms the results of our study as serotype 3 is the only serotype we have seen from stored samples of that particular outbreak of 2008.

In the present study we were able to characterize a very low number of suspected dengue samples (17.5%; 20 samples out of 114) on molecular level. This may be due to the reason that majority of samples were collected from suspected gangue virus infected patients in post viremic phase. For the correct molecular characterization of the virus, samples should be collected in acute phase of infection. Presentation of patients in post viremic phase or lower rate of viral isolation may be the reason of getting only twenty samples with positive results for dengue virus [4]. Use of serum sample for viral isolation has been advocated previously in many studies [4,7,27] so we extracted viral RNA using sera samples. The region of *C-prM *gene junction was selected for serotyping as the region is not very hyper variable and most of the mutations reported are of silent type [12]. Lifecycle of dengue virus involves both human and mosquitoes and this might be the reason for low rate of variation among dengue virus as compared to other RNA viruses. According to several reports, the classification of dengue genotypes is based on less than 6% of nucleotide divergence within a selected genomic region [12,28]. Dendrograms were drawn to study the evolutionary history of the sequenced serotypes as well as their genotypes which showed that serotype 2 circulating in 2007-2009 belonged to genotype IV. Strains from Northern India, China and Indonesia also fall in this subtype [12]. No particular pattern of genotype distribution can be inferred for serotype 2 as different genotypes spread in diverse locations. For serotype 3, only sequences of capsid region from genotype I and III are reported. So the tree was created using global sequences of genotype I and III only. However, the tree visibly shows that the studied serotype 3 has genotype III. It is clear from the findings of our study that there is no definite pattern of distribution of subtype III of dengue virus 3 worldwide [4]. The previously sequenced three strains from Karachi (Pakistan) in 2005 [20] also have same genotype emphasizing the fact that genotype III of dengue virus 3 prevails in Pakistan. There is not much data available from Pakistan on serotypes of dengue virus; this study is the first one to characterize serotypes 2 and 3 in their respective subtypes. The only limitation of this study is small number of sequenced samples. There is a need for more randomized and multi-analysis studies to be conducted on serotyping and subtyping of different dengue strains in Pakistan; in this way a clearer view on spread of dengue virus can be made.

## Conclusions

Based on the findings of the current study we conclude that the predominant serotypes of dengue virus circulating in Pakistan are 2 and 3. Ample number of cases with mixed serotypes (serotype 2 and 3) are seen and might be common in all regions of this country. The major genotypes circulated in the study period are subtype IV of dengue virus 2 and subtype III of dengue virus 3.

## Methods

### Patients Samples and Extraction of viral RNA

A total of 114 serum samples were received from Gurki Trust hospital Lahore and Sheikh Zayed Medical Complex Lahore. Viral RNA was extracted from 140 μl of serum sample using Nucleospin Viral RNA Extraction Kit (Macherey-Nagel, Germany) with slight modifications. Briefly, 600 μl of lysis buffer was added to 140 μl of serum sample and vortexed for few seconds. Then the samples were incubated at 70°C for 5 minutes. Then 600 μl of absolute alcohol was added. The sample was loaded in the column tube and centrifuged at 13000 rpm for one minute. A 500 μl of buffer RAW was added and centrifuged at 13000 rpm for 5 minutes. Then the sample was washed with 200 μl of buffer RAV3 by centrifugation at 13000 rpm for 2 minutes. Ethanol was completely removed by spinning the column for 1 minute. The column was incubated for 5 minutes at 70°C. Finally RNA was eluted in 50 μl of elution buffer and stored at -70°C till further use. The subjects gave informed consent and the study was conducted in accordance with the 1964 Declaration of Helsinki and Guidelines for Good Clinical Research Practice in Pakistan. The study was approved by Ethics Committee of Molecular Virology Division.

### Primer designing

Dengue group-specific degenerative primers were designed according to the primer sequences targeting *C-prM *gene junction described by Lanciotti *et al *[29]. Serotype-specific primers were designed using Primer3 software (Table 2). The amplified product size for specific serotypes were 411-bp for serotype-1, 403-bp for serotype-2, 453-bp for serotype-3 and 401-bp for serotype-4.

**Table 2:** Oligonucleotide sequences used to amplify C-prM gene junction of dengue virus.

**Sr. No**.	Primer Name	5'-3' Sequence	Size of amplified product in base pairs
**1**	D1-D	TCAATATGCTGAAACGCGWGAGAAACCG	**511 bp**
**2**	D2-D	TTGCACCARCARTCWATGTCTTCWGGYTC	
**3**	TS1-F	AGGACCCATGAAATTGGTGA	**411 bp**
**4**	TS1-R	ACGTCATCTGGTTCCGTCTC	
**5**	TS2-F	AGAGAAACCGCGTGTCAACT	**403 bp**
**6**	TS2-R	ATGGCCATGAGGGTACACAT	
**7**	TS3-F	ACCGTGTGTCAACTGGATCA	**453 bp**
**8**	TS3-R	CAGTAATGAGGGGGCATTTG	
9	TS4-F	CCTCAAGGGTTGGTGAAGAG	**401 bp**
	
*10*	TS4-R	CCTCACACATTTCACCCAAGT	

### Complementary DNA synthesis

Complementary DNA (cDNA) from viral RNA was synthesized using 10 μl (from 20-50 ng) of extracted RNA with a reaction mixture of 10 μl containing 4 μl 5 × First Strand Buffer, 0.5 μl 0.1 M Dithiothriotol, 2 μl 10 mM dNTPs, 1 μl 20 pM anti-sense primer and 1.3 μl dH_2_O with 0.2 μl RNase inhibitor (8 units) and 1 μl (200 units) of M-MLV Reverse Transcriptase Enzyme (Invitrogen Biotechnologies USA). The 20 μl total mixes was incubated at 37°C for 50 minutes followed by 2 minutes heat inactivation of M-MLV at 95°C. The samples were then incubated for 2 minutes at 22°C.

### Nested Polymerase Chain reaction

Nested PCR was used for serotyping analysis of samples. For amplification of cDNA, 5 μl of cDNA (50-100 ng) was used with 15 μl of PCR mix containing 2 μl 10 × PCR Buffer, 2.4 μl MgCl_2 _(from 25 mM stock), 1 μl 500 μM dNTPs, 1 μl 20 pM forward and reverse primer each, 5.6 μl dH_2_O and 2 unites of *Taq*-DNA polymerase enzyme (Invitrogen Biotechnologies USA). The thermal profile for first round (using outer sense D1-D and anti-sense D2-D) was: initial denaturation at 94°C for 2 minutes followed by 35 cycles of denaturation at 94°C for 45 seconds, annealing at 52°C for 45 seconds and extension at 72°C for 2 minutes. A final extension was given at 72°C for 10 minutes. The thermal profile for second round using the type-specific sense and anti-sense primers was same to the thermal profile of first round, only the annealing was carried out at 54°C for 45 seconds in 35 cycles.

### Gel elution

The PCR product (nested PCR) was run on 2% agarose gel. Gel purification was done using the QIAquick Gel Extraction Kit (Qiagen, Germany) and the purified product was eluted in 30 μl of double distilled water which was used as a template for sequencing reaction

### Sequencing reaction and ethanol precipitation

Sequencing analysis was performed on automated genetic analyser according to the manufacturer's instructions (Big Dye Deoxy Terminators; Applied Biosystems, Weiterstadt, Germany). Concentration of 25 ng of eluted DNA was used for sequencing PCR reaction. Briefly, 10 μl reaction mixture was prepared using 0.6 μl of BigDye, 1.5 μl 5× sequencing buffer, 1.5 μl (about 25 ng) template, 1 μl 20 pM sense or anti-sense primer and 5.4 μl of dH_2_O. The amplification steps in thermal cycler were: initial denaturation at 96°C for one minute followed by 35 cycles of denaturation at 96°C for 15 seconds, annealing at 50°C for 10 seconds and extension at 60°C for 4 minutes. Final extension was given at 60°C for 4 minutes. Ethanol precipitation of sequencing PCR product was carried out by adding 2 μl 3 M sodium acetate, 2 μl 125 mM ethylenediaminetetraacetic acid (EDTA) and 26 μl of absolute alcohol. The mixture was put at room temperature for 15-20 minutes. It was centrifuged for 30 minutes at 3800 rpm at 4°C. Thirty-six micro litres of 70% ethanol was added to dry pellet and centrifuged for 15 minutes at 3800 rpm. Finally 12 μl of formamide was added to dried pellet and mixed well. It was followed by heat shock at 95°C for 5 minutes and was loaded onto automated sequencer (Applied Biosystems, 3100 DNA Analyzer) for sequence analysis.

### Nucleotide and amino acid sequence analysis

The obtained sequences were edited and BLAST search was conducted to confirm the identity of the sequences. The amino acid sequences were translated using BioEdit v7.0.5 software and also it was used to align amino acid and protein sequences. The phylogenetic and molecular evolutionary analyses were conducted using MEGA version 4 [30]. The phylogenetic tree was drawn by using the Neighbor-Joining method with bootstrap analysis of 1000 replicates. The sequences of different geographical regions were retrieved from GenBank and their accession numbers for sequences of serotype 2 and serotype 3 appear in Figures 1 and 2.

## Competing interests

The authors declare that they have no competing interests.

## Authors' contributions

MI conceived of the study, participated in its design and coordination and gave a critical view of manuscript writing. ZF performed, sequenced and analyzed the results. MAB, ZT, OU AND MQZ helped ZF in sample collections. MA, AH, BK, SA, SM, SS, BR, SB, MN, SB, MA, LA and MA participated in analysis of results and manuscript writing. All the authors read and approved the final manuscript.
